# Human stem cell-derived β cells expressing an optimized CD155 reduce cytotoxic immune cell function for application in type 1 diabetes

**DOI:** 10.1126/sciadv.adx9755

**Published:** 2025-11-07

**Authors:** Matthew E. Brown, Jessie M. Barra, Marcus R. Pina, James Proia, Todd M. Brusko, Holger A. Russ

**Affiliations:** ^1^ Diabetes Institute, University of Florida, Gainesville, FL, USA.; ^2^ Department of Pathology, Immunology and Laboratory Medicine, College of Medicine, University of Florida, Gainesville, FL, USA.; ^3^ Department of Pharmacology and Therapeutics, College of Medicine, University of Florida, Gainesville, FL, USA.; ^4^ Department of Pediatrics, College of Medicine, University of Florida, Gainesville, FL, USA.; ^5^ Department of Biochemistry and Molecular Biology, College of Medicine, University of Florida, Gainesville, FL, USA.

## Abstract

Insulin-producing β cell replacement therapies show promise for treating type 1 diabetes (T1D), but challenges such as donor shortages and immune rejection persist. Stem cell–derived β cells (sBC) provide a renewable source but remain susceptible to immune attack. We engineered human pluripotent stem cells to express either the wild type (WT) or a high-affinity mutant (Mut) variant (rs1058402, G>A; Ala^67^Thr) of the natural killer (NK) and T cell checkpoint inhibitor CD155 before differentiation into sBC. Modified sBC maintained up-regulated CD155 expression and showed enhanced binding to co-receptor ligands. Co-culture studies revealed CD155-expressing sBC suppressed autoreactive CD8^+^ T cell and NK cell activation, reducing immune cell–mediated sBC destruction and cytotoxic molecule secretion by preferentially engaging the coinhibitory receptor TIGIT. This protection was lost with TIGIT blockade, affirming the role of CD155-TIGIT signaling in antagonizing immune cell cytotoxicity. Our findings suggest that high-affinity CD155 expression enhances immune evasion of sBC, improving their potential as a therapy for T1D.

## Teaser

Engineered β cells with a mutant CD155 help evade immune attack, offering a promising therapeutic approach for type 1 diabetes.

## INTRODUCTION

Pancreatic β cell replacement strategies hold great promise as a treatment option for patients with type 1 diabetes (T1D). However, challenges for the widespread implementation of this potentially curative approach include limited cadaveric donor tissue availability, immediate side-effects and long-term complications of systemic immune suppression, and cellular stress- or immune-mediated graft failure in a majority of patients by 5 years postinfusion (*1*–*5*). The differentiation of human stem cell–derived β cells (sBC) from human pluripotent stem cells (hPSCs) negates the requirement for organ donation, yielding a potentially limitless pool of human β cells for clinical use (*6*, *7*). Improvements in differentiation protocols over the past decade now allow for the generation of functional, glucose-responsive sBC that can restore glycemic control in preclinical animal models (*8*–*14*) and in an ongoing first phase I/II clinical trial in human patients with T1D (clinicaltrials.gov; NCT04786262).

Still, similar to cadaveric islets, sBC are susceptible to immune-mediated destruction upon transplantation, necessitating the use of systemic immunosuppressive drugs, thereby excluding a larger patient population, due to the potential risk of serious secondary complications, such as viral infections, nephrotoxicity, and neurotoxicity (*15*, *16*). To enhance the widespread implementation of sBC therapies, alternative approaches to prevent immune-mediated graft destruction are essential. Patients with T1D can mount two unique immune cell responses toward transplanted β cells: (i) allogeneic rejection of the immunologically mismatched tissues and (ii) a recurrence of autoimmunity toward β cell autoantigens. Both immune responses must be efficiently suppressed to maintain the long-term survival and function of any sBC graft.

With advances in genome engineering, hPSC can now be effectively modified to modulate the expression of key immune regulatory genes before differentiation into sBC (*17*–*22*). Multiple groups have demonstrated that this approach is efficacious in suppressing allogeneic and potentially even xenogeneic immune responses toward transplanted sBC in preclinical models (*17*, *20*, *23*). However, there is limited understanding of whether any modification can withstand autoimmune attacks, largely due to the lack of appropriate human model systems.

The immune checkpoint ligand and adhesion molecule, CD155 [poliovirus receptor (PVR)], modulates immune responses through interactions with the natural killer (NK) cell and T cell coinhibitory receptor, TIGIT (T cell immunoreceptor with Ig and ITIM-like domains) (*24*). Although CD155 can also participate with CD226 [DNAM-1; *K*_d_ (dissociation constant) = 119 nM] and CD96 (TACTILE; *K*_d_ = 37.6 nM) to promote costimulatory and coinhibitory signaling, respectively, CD155 preferentially interacts with TIGIT (*K*_d_ = 3.15 nM) to restrain T cell activation (*25*).

In the context of autoimmunity, there has been increased interest in leveraging this signaling axis to restore immune tolerance by skewing NK cells and T cells away from CD155/CD226 signaling and toward CD155/TIGIT signaling, given the presence of a T1D risk-associated single-nucleotide polymorphism (SNP) in *CD226* (rs763361; C>T), thought to enhance downstream signaling (*26*). We demonstrated that a conditional knockout (cKO) of *Cd226* in regulatory T cells (T_reg_ cells) reduces disease incidence in the nonobese diabetic (NOD) model of autoimmune diabetes by increasing the expression of TIGIT on pancreatic CD4^+^ T cells (*27*) and, most recently, that monoclonal antibody (mAb) blockade of CD226 reduces disease incidence in the NOD by enhancing the immunoregulatory function of T_reg_ cells and inhibiting the function of proinflammatory effector T cells (*28*). We have recently reported that pancreatic β cells exhibit reduced expression of CD155 during T1D pathogenesis, suggesting dysregulation within this signaling pathway (*29*). Further, approaches seeking to bolster CD155/TIGIT signaling to limit T cell activity have shown promise in the treatment of autoimmunity, including the use of recombinant TIGIT-immunoglobulin (TIGIT-Ig) fusion proteins to prolong survival in the NZB/W F1 mouse model of lupus (*30*) and the expression of TIGIT on CD4^+^ T cells using lentivirus to reduce collagen-induced rheumatoid arthritis (RA) in a BALB/C mouse model (*31*).

In oncology, the CD155/TIGIT checkpoint has been characterized as a critical mechanism for tumor immune escape by disrupting costimulatory signaling (*32*) and inducing a loss of effector function in tumor-infiltrating T cells. Consistent with these findings, the expression of a high-affinity mutant (rs1058402; G>A; Ala^67^Thr) of CD155 in patients with small cell lung cancer demonstrated poorer treatment outcomes (*33*). Biolayer interferometry (BLI) experiments revealed that this mutant CD155 exhibits greater binding affinity toward both TIGIT and CD226 (*34*). Furthermore, NK cells cocultured with a mutant CD155 (CD155 Mut)–expressing epithelial line exhibited reduced activation compared to NK cells cocultured with the WT CD155 (CD155 WT)–expressing epithelial line (*34*).

To understand whether the expression of either the CD155 Mut or CD155 WT could reduce allogenic and antigen-specific, autoreactive T cell responses and NK cell responses, we differentiated genome-edited hPSC lines into functional sBC and performed detailed mechanistic in vitro coculture studies with islet antigen-reactive primary human T cells and NK cells. We investigated the immunogenicity of each human sBC line and how the modulation of the CD155 signaling pathway may confer protection from allo- and autoimmunity to better inform future pancreatic β cell replacement therapies.

## RESULTS

### Generation of WT and Mut CD155 hPSC Lines

**Fig. 1.**
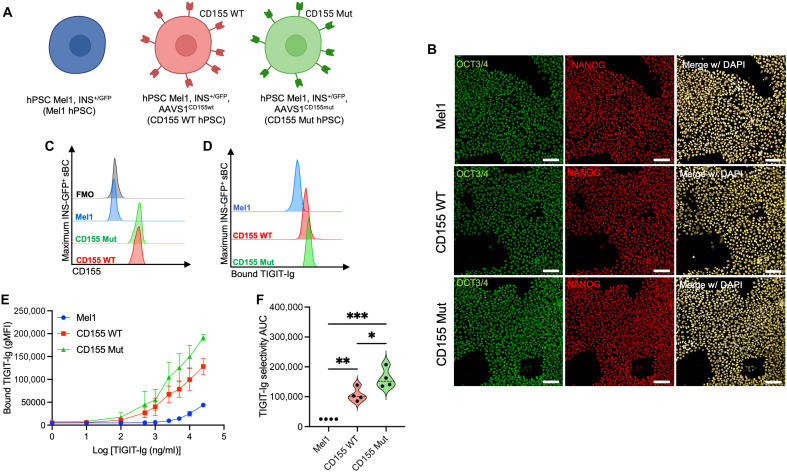
Genome engineering of CD155 WT and CD155 Mut Expressing hPSC. (**A**) Schematic of parental Mel1^INS-GFP^ (blue) and engineered WT (red) or Mut CD155 (green) hPSC lines [Created in BioRender. Russ, H. (2025); https://BioRender.com/r06dcp3]. (**B**) Immunofluorescence staining for OCT3/4 (green), NANOG (red), and 4′,6-diamidino-2-phenylindole (white) in hPSC. Scale bar represents 100 µm. (**C**) Flow cytometry for expression of CD155 in hPSC. (**D** to **F**) hPSC were treated with fluorescently labeled TIGIT-Ig to compare binding efficiency between the parental Mel1 (blue), CD155 WT–expressing (red), and CD155 Mut–expressing (green) lines. (D) Representative histograms of cell-bound Ig after treatment at the 25,000 ng/ml condition. (E) Differences in Ig binding efficiency characterized across a range of 0 to 25,000 ng/ml, with additional comparisons made using (F) TIGIT-Ig selectivity area under the curve (AUC) values for each binding curve, where the bound TIGIT-Ig gMFI value for each line was normalized to the Mel1-bound TIGIT-Ig gMFI at each concentration. Data reflect biological *n* = 3 per condition for TIGIT-Ig sBC. Significant *P* values are reported for one-way analysis of variance (ANOVA) with Bonferroni’s multiple comparisons of AUC values between paired samples. *P < 0.05, **P < 0.01, and ***P < 0.001.

To test whether CD155 expression can be used to reduce or prevent T cell activation by sBC, we introduced a genetically encoded expression cassette into the hPSC line Mel1^INS-GFP^, which contains a green fluorescent protein (GFP) reporter gene driven by the endogenous insulin promoter (pINS.GFP) (*35*). Using established protocols for TALEN-mediated site-specific genetic engineering of the AAVS1 locus in hPSC (*19*, *20*), we targeted Mel1^INS-GFP^ hPSC to constitutively express WT or Mut CD155 in (Fig. 1A and fig. S1B) and confirmed that targeting did not negatively affect the overt hPSC morphology of any isolated clonal hPSC line. Furthermore, the protein expression of pluripotency markers OCT3/4 and NANOG was readily detectable by immunofluorescence (IF) staining (Fig. 1B). The successful modification of clonal colonies was evaluated via flow cytometry to assess expression of CD155 (Fig. 1C).

To characterize if the CD155 WT or Mut expression influenced the binding of the ligand TIGIT, we cultured hPSC with fluorescently labeled TIGIT-Ig fusion protein. Across a range of 0 to 25,000 ng/ml, we observed that both CD155 WT and CD155 Mut hPSC displayed greater binding efficiency for TIGIT-Ig (Fig. 1, D to F; CD155 WT: 4.25-fold, *P* = 0.0054; CD155 Mut:6.47-fold, *P* = 0.0003) compared to the parental Mel1 hPSC. Notably, the CD155 Mut line displayed greater cell binding for TIGIT-Ig (1.52-fold, *P* = 0.033) compared to the CD155 WT line. These differences were not observed with a nonspecific isotype control Ig antibody (fig. S2A).

### CD155 expressing hPSC efficiently differentiate into sBC

**Fig. 2.**
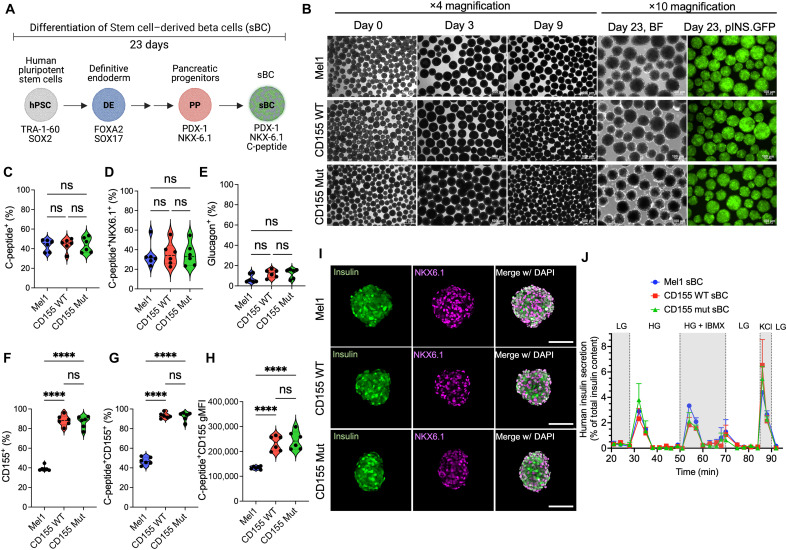
CD155 WT and CD155 Mut sBC show similar differentiation and function. (**A**) Schematic of differentiation from hPSC to sBC [Created in BioRender. Russ, H. (2025); https://BioRender.com/0d823q8]. (**B**) Representative bright field and pINS.GFP reporter live images of differentiating clusters at subsequent time points. ×4 or ×10 magnification images as denoted. (**C** to **H**) Flow cytometry analysis at day 23 of *n* = 6 independent differentiations of Mel1, CD155 WT, and CD155 Mutant sBC for frequency of (C) C-peptide^+^ cells, (D) C-peptide^+^ NKX6.1^+^ double-positive cells, (E) glucagon^+^ cells, (F) total CD155^+^ cells or (G) CD155^+^ C-peptide^+^ populations, and (H) geometric mean fluorescence intensity (gMFI) of CD155 within C-peptide^+^ cells. (**I**) Immunofluorescence staining of day 23 sBC for insulin (green), NKX6.1 (magenta), and DAPI (white). Scale bar represents 200 µm. (**J**) Dynamic glucose stimulated insulin secretion data from perfusion study as percent of total insulin content. *n* = 3 independent differentiations. Significant P-values are reported for one-way ANOVA with Bonferroni’s multiple comparisons. ****P < 0.0001. ns, not significant; IBMX, 3-isobutyl-1-methylxanthine (IBMX); PP, pancreatic progenitors; DE, definitive endoderm.

Using our previously established methods (*36*), we then performed directed differentiation into sBC experiments with both modified hPSC lines and the unmodified parental hPSC line as a control (Fig. 2A). Flow cytometric analysis for pluripotency markers TRA-1-60 and SOX2 demonstrate uniform expression of pluripotency markers in all three hPSC lines at the start of differentiation, which was lost after definitive endoderm (DE) generation at day three, supporting the IF data. As expected, DE markers FOXA2 and SOX17 were absent at the start but showed high expression by the third day of differentiation (fig. S1, G to J). At subsequent days of differentiation, the morphology of Mel1, CD155 WT, and CD155 Mut hPSC clusters displayed similar morphology and expression of the pINS.GFP reporter at the end of differentiation (Fig. 2B). At day 23 of the protocol, all hPSC lines reproducibly generated ~50% insulin-expressing sBC. We did not observe statistical significant differences in the frequency of β cell markers C-peptide (Fig. 2C) and NKX6.1 (Fig. 2D) or the frequency of alpha cell marker glucagon (Fig. 2E) by flow cytometry. However, when we measured the expression of CD155 on sBC, we observed the retention of CD155 expression in virtually all the modified cells whether assessing total population (Fig. 2F) or in C-peptide^+^ sBC cells (Fig. 2G). The frequencies and the geometric mean fluorescence intensity (gMFI) of CD155 expression were significantly higher in edited sBC compared to Mel1 sBC (Fig. 2H), similar to our hPSC analysis for transgene expression. IF staining of day 23 sBC revealed comparable morphology and expression of insulin and NKX6.1 across all three lines (Fig. 2I). To assess the sBC function, we conducted dynamic glucose-stimulated insulin secretion (GSIS) assays and demonstrated that CD155 WT and CD155 Mut sBC are similarly responsive to high glucose, 3-isobutyl-1-methylxanthine (IBMX) (triggering the amplifying pathway) (*37*), and depolarization with KCl compared to Mel1 sBC controls (Fig. 2J). Collectively, these data demonstrate that expression of CD155 does not influence the differentiation efficiency or function of sBC.

### Mutant CD155 exhibits greater binding for TIGIT-Ig and CD226-Ig

**Fig. 3.**
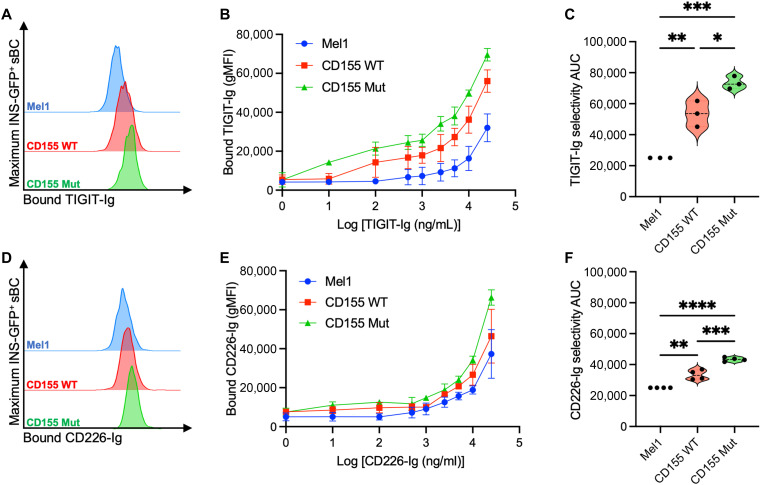
Expression of mutant CD155 confers greater binding for TIGIT-Ig and CD226-Ig on sBC. sBC were treated with fluorescently labeled (**A** to **C**) TIGIT-Ig or (**D** to **F**) CD226-Ig to compare binding efficiency between the parental Mel1 (blue), CD155 WT–expressing (red), and CD155 Mut–expressing (green) lines. [(A) and (D)] Representative histograms show cell-bound Ig after treatment at the 25,000 ng/ml condition. [(B) and (E)] Differences in Ig binding efficiency were characterized across a range of 0 to 25,000 ng/ml, with additional comparisons made using [(C) and (F)] AUC values for each binding curve. Data reflect biological *n* = 3 per condition for TIGIT-Ig sBC and *n* = 4 per condition for CD226-Ig. Significant *P* values are reported for one-way ANOVA with Bonferroni’s multiple comparisons of AUC values between paired samples. *P < 0.05, **P < 0.01, ***P < 0.001, and ****P < 0.0001.

To characterize how the expression of either WT or Mut CD155 on sBC impacted binding toward TIGIT or CD226, we cultured sBC with fluorescently labeled TIGIT-Ig fusion protein. Across a range of 0 to 25,000 ng/ml, we observed that both CD155 WT and CD155 Mut sBC displayed greater binding efficiency for TIGIT-Ig (CD155 WT: 2.14-fold, *P* = 0.0064; CD155 Mut: 2.93-fold, *P* = 0.0008; Fig. 3, A to C) and CD226-Ig (CD155 WT: 1.33-fold, *P* = 0.0013; CD155 Mut: 1.73-fold, *P* < 0.0001; Fig. 3, D to F) compared to the parental Mel1 sBC. Notably, the CD155 Mut line displayed greater binding for both TIGIT-Ig (1.37-fold, *P* = 0.024) and CD226-Ig (1.29-fold, *P* = 0.0005) as compared to the CD155 WT line. Using a control isotype Ig in the same assay demonstrated no significant differences between the three lines (fig. S2B). These data support previously reported BLI results by Matsuo *et al.* (*34*), suggesting that Mut CD155 demonstrates a greater efficiency for the ligands TIGIT and CD226.

### IFN-γ does not up-regulate CD112 or CD155 expression on sBC

**Fig. 4.**
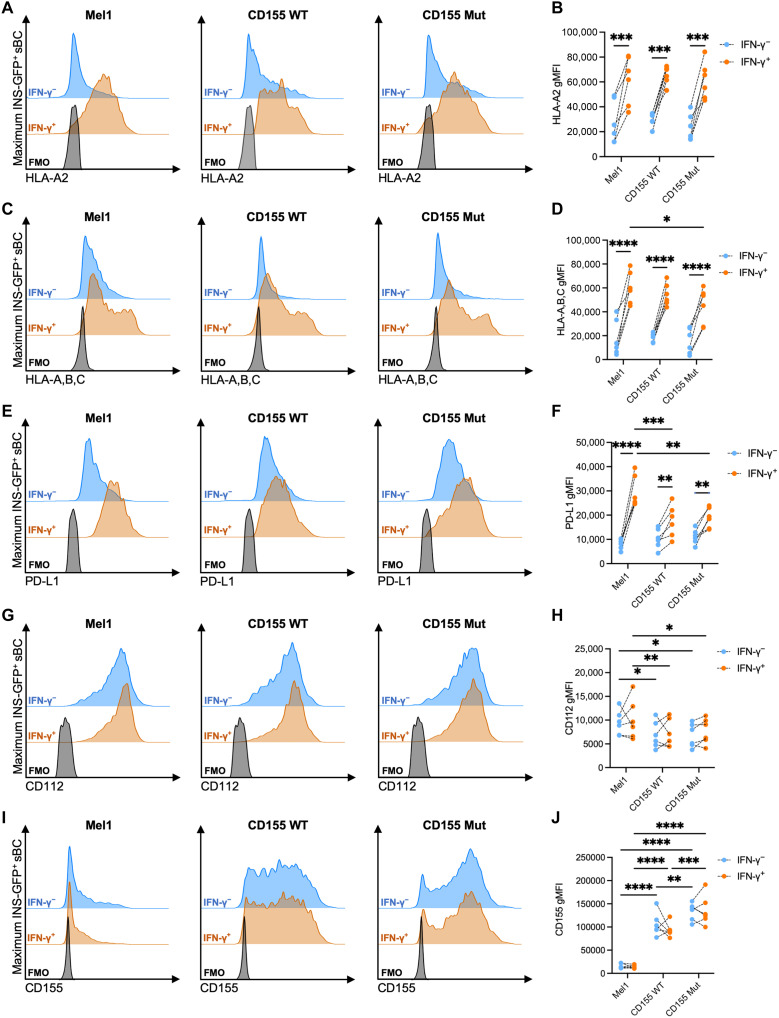
IFN-γ treatment does not up-regulate CD112 or CD155 expression on sBC. The expression of (**A** and **B**) HLA-A2; (**C** and **D**) HLA-A,B,C; (**E** and **F**) PD-L1; (**G** and **H**) CD112; and (**I** and **J**) CD155 was evaluated on INS-GFP^+^ sBC following 48 hours with (orange) or without (blue) IFN-γ treatment. [(A), (C), (E), (G), and (I)] Representative histograms show staining of Mel1, CD155-WT, and CD155-Mut sBC lines relative to fluorescence-minus one (FMO) controls (black). [(B), (D), (F), (H), and (J)] Paired dot plots show gMFI values by sBC line and treatment condition. Data reflect biological *n* = 6 per condition. Significant *P* values are reported for two-way ANOVA with Bonferroni’s multiple comparisons between paired samples. *P < 0.05, **P < 0.01, ***P < 0.001, and ****P < 0.0001.

In the inflamed pancreatic islet microenvironment during the pathogenesis of T1D, interferon-γ (IFN-γ) has been shown to promote immunogenicity through up-regulating major histocompatibility complex–I (MHC-I) molecules (*38*, *39*). We observed greater expression of HLA-A2 (Fig. 4, A and B) and HLA-A,B,C (Fig. 4, C and D) following IFN-γ treatment across all three sBC lines and up-regulation of programmed cell death ligand 1 (PD-L1) (Fig. 4, E and F) as we have previously reported (*19*). These data indicate that CD155 genome engineering does not negate prototypical mechanisms of standard immune surveillance or response to cytokine treatment, which would be a caveat of any immunological assessments. However, we did observe reductions in CD112 (Fig. 4, G and H) expression, another ligand that can bind TIGIT and CD226 (*40*), on CD155-expressing sBC compared to Mel1 controls and small but significant reductions in HLA-A,B,C and PD-L1 in IFN-γ–treated conditions. The very high levels of CD155 expression driven by the CAG promoter in our system may be limiting available cellular surface receptor space causing reductions in some receptor gMFI upon IFN-γ stimulation (*41*). However, as modified sBC are still capable of up-regulating non-CD155 surface receptor expression upon IFN-γ stimulation, the observed small differences may not have biological significance. Nonetheless, we did not detect a significant impact on the expression of CD112 or CD155 (Fig. 4, I and J) after IFN-γ treatment, suggesting that the expression of these ligands is not affected by the cytokine treatment.

### CD8^+^ T cells demonstrate reduced activation following coculture with CD155 Mut sBC

**Fig. 5.**
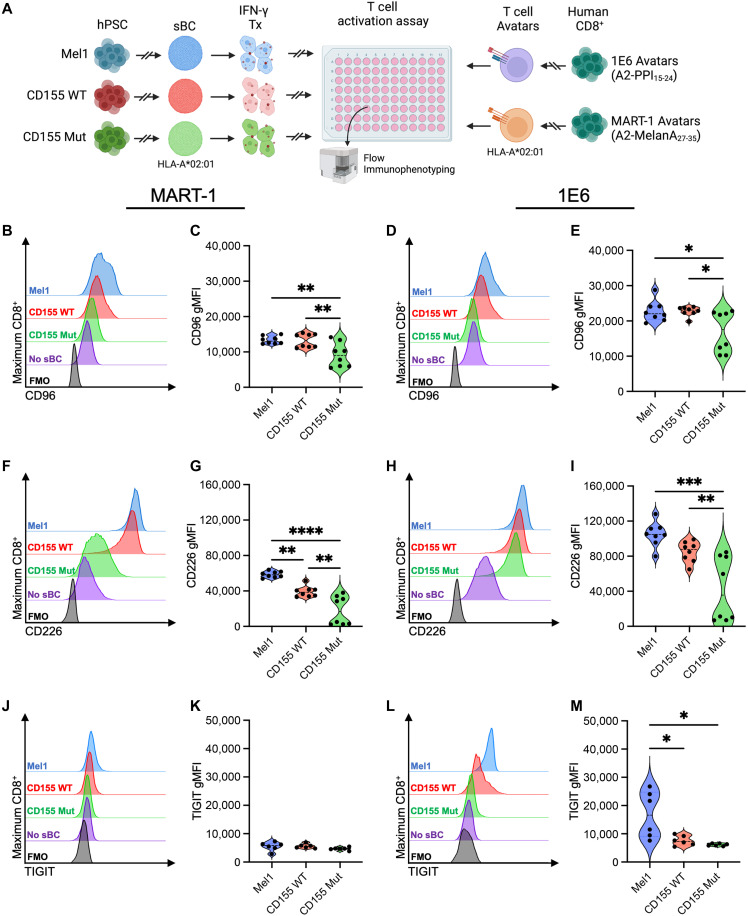
Expression of mutant CD155 reduces T cell activation during HLA-peptide-TCR–matched coculture. (**A**) Experimental scheme depicting activation assay to assess the immunogenicity of Mel1 (blue), CD155 WT–expressing (red), and CD155 Mut–expressing (green) sBC by MART- or PPI-reactive avatars, as measured by flow immunophenotyping of T cells [Created in BioRender. Brown, M. (2025); https://BioRender.com/sdjn41g]. Plots show differences in expression of the T cell activation markers, (**B** to **E**) CD96, (**F** to **I**) CD226, and (**J** to **M**) TIGIT on [(B) and (C), (F) and (G), and (J) and (K)] MART-1– or [(D) and (E), (H) and (I), and (L) and (M)] PPI-reactive avatars between sBC lines at the 10:1 E:T ratio compared to no sBC (purple) and no dye (black) controls. Data reflect biological *n* = 8 per condition. Significant *P* values are reported for one-way ANOVA with Bonferroni correction for multiple comparisons. *P < 0.05, **P < 0.01, ***P < 0.001, and ****P < 0.0001.

To understand whether expression of the WT or Mut CD155 on sBC affects the activation of responder CD8^+^ T cells, we performed human leukocyte antigen (HLA)–peptide–T cell receptor (TCR)–matched coculture assays with T cell avatars specific for an irrelevant antigen (clone MART-1) or toward preproinsulin (PPI) (clone 1E6), a canonical autoantigen presented to T cells by sBC at an effector:target (E:T) ratio of 10:1 (Fig. 5A). Following 48 hours of coculture with CD155 Mut sBC, when examining the expression of the CD155 ligands, we observed that MART-1– and PPI-reactive avatars cocultured with CD155 Mut sBC displayed reduced expression of CD96 (Fig. 5, B to E) compared to avatars cocultured with CD155 WT (MART-1: 0.69-fold, *P* = 0.0025; PPI: 0.74-fold, *P* = 0.031) or Mel1 (MART-1: 0.69-fold, *P* = 0.0019; PPI: 0.74-fold, *P* = 0.031) sBC with even more apparent reductions in CD226 expression (Fig. 5, F to I) compared to avatars cocultured with CD155 WT (MART-1: 0.46-fold, *P* = 0.0024; PPI: 0.50-fold, *P* = 0.0097) or Mel1 (MART-1: 0.31-fold, *P* < 0.0001; PPI: 0.40-fold, *P* = 0.0004) sBC. MART-1 T cell avatars demonstrated similar levels of TIGIT expression after coculture with the different sBC (Fig. 5, J and K), while PPI-reactive avatars displayed a significant reduction in TIGIT expression after coculture with CD155-engineered sBC (Fig. 5, L and M), suggesting a potential antigen-specific difference in this pathway. These data suggest that the expression of mutant CD155 by sBC reduces the activation and functionality of CD8^+^ T cells following engagement.

**Fig. 6.**
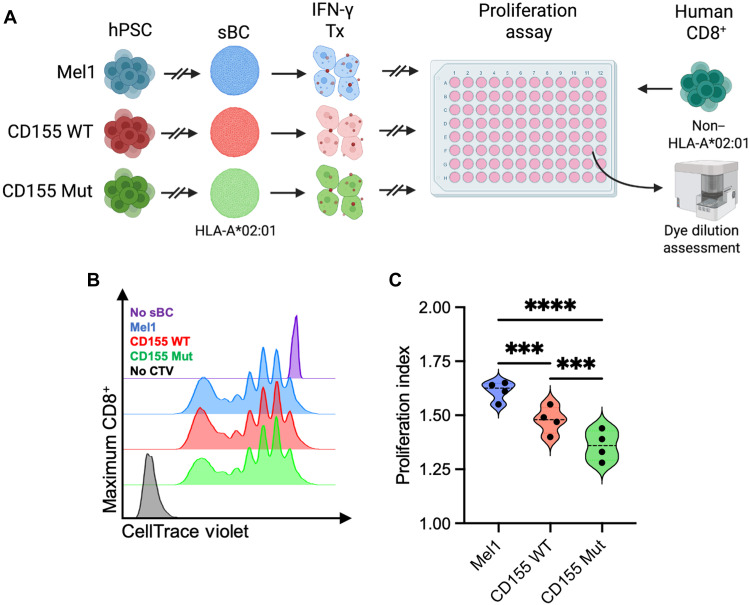
Expression of mutant CD155 reduces CD8^+^ T cell allogeneic proliferative responses. (**A**) Experimental scheme depicting proliferation assay used to characterize the immunogenicity of sBC lines when cultured with allogenic naïve CD8^+^ T cells [Created in BioRender. Brown, M. (2025); https://BioRender.com/5fgeu0g]. (**B**) Representative dye dilution plots depict the proliferation of naïve CD8^+^ T cells following 6 days of coculture with Mel1 (blue), CD155 WT–expressing (red), and CD155 Mut–expressing (green) sBC compared to no sBC (purple) and no dye (black) controls, with (**C**) violin plots showing proliferation indices for each condition (biological *n* = 4 per condition). Significant *P* values are reported for paired samples using one-way ANOVA with Bonferroni correction for multiple comparisons. ***P < 0.001 and ****P < 0.0001.

To determine how the expression of Mut CD155 affects allogeneic T cell responses, we cultured proliferation dye-labeled naïve CD8^+^ T cells with sBC to assess proliferative capacity (Fig. 6A). Following 6 days of coculture, we observed reductions in CD8^+^ T cell proliferation when cultured with CD155 WT (0.92-fold, *P* = 0.0003) or CD155 Mut (0.84-fold, *P* < 0.0001) sBC compared to the parental Mel1 sBC (Fig. 6, B and C). Furthermore, we identified greater reductions in CD8^+^ T cell proliferation in the CD155 Mut coculture (0.92-fold, *P* = 0.0006) compared to the CD155 WT coculture (Fig. 6, B and C). Together, these data suggest that the expression of CD155, particularly the Mut CD155, by sBC reduces immunogenicity from alloreactive T cells.

### Expression of CD155 Mut confers greater resistance to sBC from T cell–mediated lysis

**Fig. 7.**
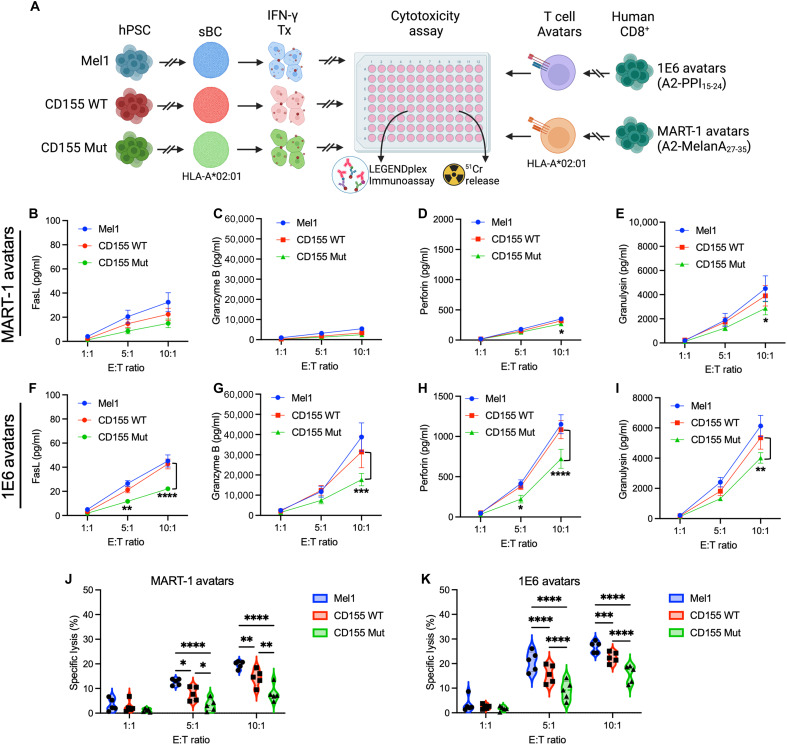
Expression of mutant CD155 provides sBC greater protection from T cell–mediated lysis. (**A**) Experimental scheme depicting CML assay to assess the immunogenicity of Mel1 (blue), CD155 WT–expressing (red), and CD155 Mut–expressing (green) sBC by MART- or PPI-reactive avatars, as measured by ^51^Cr release, with T cell cytokine production assessed by LEGENDplex [Created in BioRender. Brown, M. (2025); https://BioRender.com/nehgyik]. Plots show differences in cell culture supernatant concentrations following coculture with (**B** to **E**) MART-1– or (**F** to **I**) PPI-reactive avatars of [(B) and (F)] FasL, [(C) and (G)] granzyme B, [(D) and (H)] perforin, and [(E) and (I)] granulysin between sBC lines at each E:T ratio. Data reflect biological *n* = 8 per condition. Significant *P* values are reported for two-way ANOVA with Bonferroni correction for multiple comparisons between CD155 WT and CD155 Mut conditions. (**J** and **K**) Violin plots show percent-specific lysis of sBC by (J) MART-1– or (K) PPI-reactive avatars at each E:T ratio. Data reflect biological *n* = 5 per condition. Significant *P* values are reported for two-way ANOVA with Bonferroni correction for multiple comparisons. *P < 0.05, **P < 0.01, ***P < 0.001, and ****P < 0.0001.

To understand how the expression of either the WT or Mut CD155 affects the cytotoxicity of T cells toward sBC, we performed coculture assays with MART-1 and PPI avatars (Fig. 7A) at varying E:T ratios of: 1:1, 1:5, and 1:10. After 2 days of coculture with MART-1 avatars, we observed no significant changes in the cytolytic molecules FasL or granzyme B production between the CD155 WT and the CD155 Mut sBC lines (Fig. 7, B and C). However, at the 10:1 ratio, we observed that MART-1 avatars cocultured with CD155 Mut sBC produced significantly less perforin (0.85-fold, *P* = 0.034) and granulysin (0.74-fold, *P* = 0.032) than avatars cocultured with CD155 WT sBC (Fig. 7, D and E), suggesting that the expression of Mut CD155 can reduce T cell cytotoxicity without antigen recognition. Further, in the context of antigen-specific recognition, we observed that PPI-reactive avatars cultured with CD155 Mut sBC at a 10:1 ratio produced significantly less FasL (0.52-fold, *P* < 0.0001), granzyme B (0.56-fold, *P* = 0.0003), perforin (0.66-fold, *P* < 0.0001), and granulysin (0.75-fold, *P* = 0.0013) compared to PPI-reactive avatars cocultured with CD155 WT sBC (Fig. 7, F to I). Notably, we also observed significant decreases in the production of FasL (0.44-fold, *P* < 0.0001) and perforin (0.91-fold, *P* = 0.037) by PPI-reactive avatars cultured with CD155 Mut sBC at the 5:1 E:T ratio compared to cocultures with CD155 WT sBC.

To determine whether these decreases in immunogenicity between the CD155 WT and CD155 Mut sBC lines translate to protection from cell-mediated lysis (CML), we assessed sBC killing by T cell avatars via chromium release assay. After 48 hours of coculture, we identified that CD155 Mut sBC showed reductions in specific lysis at the 5:1 (0.42-fold, *P* = 0.021) and 10:1 (0.56-fold, *P* = 0.0015) E:T ratios following coculture with MART-1 avatars, as compared to CD155 WT sBC (Fig. 7J). Strikingly, we observed that CD155 Mut sBC also demonstrated increased protection from antigen-specific CML by PPI-reactive avatars, with significantly diminished lysis at the 5:1 (0.58-fold, *P* < 0.0001) and 10:1 (0.71-fold, *P* < 0.0001) E:T ratios compared to CD155 WT sBC (Fig. 7K).

### TIGIT blockade ablates protection conferred by CD155 expression on sBC

**Fig. 8.**
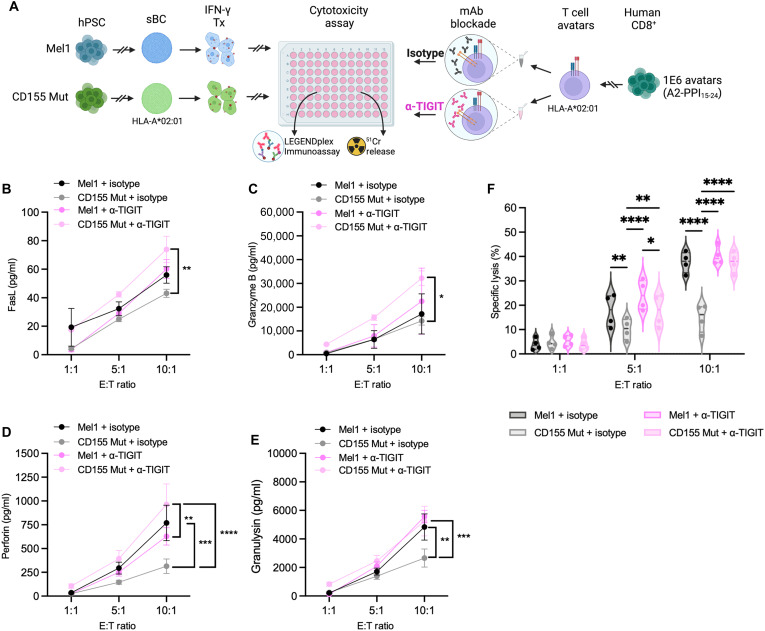
TIGIT blockade ablates sBC protection from CML conferred by Mut CD155 expression. (**A**) Experimental scheme depicting CML assay to assess the effect of TIGIT blockade (pink), relative to an isotype control (black) on CML of parental Mel1 and CD155 Mut–expressing sBC lines by PPI-reactive avatars, as measured by ^51^Cr release, with T cell cytokine production assessed by LEGENDplex [Created in BioRender. Brown, M. (2025); https://BioRender.com/3l30agq]. Plots show differences in cell culture supernatant concentrations following coculture with Mel1 or CD155 Mut sBC of (**B**) FasL, (**C**) granzyme B, (**D**) perforin, and (**E**) granulysin between anti (α)–TIGIT and isotype-treated PPI-reactive avatars at each E:T ratio. Data reflect biological *n* = 4 per condition. (**F**) Violin plots show percent-specific lysis of Mel1 (dark gray and dark pink) or CD155 Mut (light gray and light pink) sBC at each E:T ratio with or without anti-TIGIT blockade. Data reflect biological *n* = 4 per condition. Significant *P* values are reported for three-way ANOVA with Bonferroni correction for multiple comparisons. *P < 0.05, **P < 0.01, ***P < 0.001, and ****P < 0.0001.

Given the increased protection from antigen-specific CML conferred to sBC with increased availability and higher affinity CD155 Mut, we sought to determine the mechanism accounting for the reduced cytotoxic activity of CD8^+^ T cell avatars. To accomplish this, we evaluated the contribution of CD155:TIGIT signaling by treating PPI-reactive avatar T cells with an anti-TIGIT blocking antibody before coculture with Mel1 or CD155 Mut sBC (Fig. 8A). Compared to an isotype control, we did not observe significant differences in T cell cytolytic molecule production resulting from TIGIT blockade when PPI-reactive avatars were cocultured with Mel1 sBC (Fig. 8, B to E, gray lines), which would not be expected due to low CD155:TIGIT signaling. However, when TIGIT was blocked on PPI-reactive avatars and cocultured with CD155 Mut sBC (Fig. 8, B to E, pink lines), we observed greater production of the cytolytic molecules FasL (Fig. 8B; 10:1: 1.71-fold, *P* = 0.0035), granzyme B (Fig. 8C; 10:1: 2.43-fold, *P* = 0.017), perforin (Fig. 8D; 10:1: 3.06-fold, *P* < 0.0001), and granulysin (Fig. 8E; 10:1: 1.97-fold, *P* = 0.0002) compared to PPI-reactive avatars treated with an isotype control.

When assessing sBC killing by chromium-release assays, we again observed a protective impact of CD155 Mut expression on sBC compared to Mel1 control sBC cultures in the presence of the isotype antibody blockade (Fig. 8J, gray bars). Upon TIGIT blockade, we did not see any significant changes in the susceptibility of Mel1 sBC to CML (Fig. 8F, dark gray to dark pink bars), but we observed significantly increased killing of CD155 Mut sBC to CML as a result of TIGIT blockade at both the 5:1 (1.80-fold, *P* = 0.0058) and 10:1 (1.91-fold, *P* < 0.0001) E:T ratios (Fig. 8F, light gray to light pink bars). TIGIT blockade returned CD155 Mut sBC CML levels to those equivalent to Mel1 control sBC cocultures. Together, these data suggest that the increased protection conferred to sBC from antigen-specific CML by the expression of Mut CD155 is a result of more robust coinhibitory CD155:TIGIT signaling, the loss of which likely skews T cells toward preferential CD226:CD155 costimulation and augmented cytotoxic potential.

### CD155 expression in sBC suppresses NK activation and NK-mediated cytotoxicity

**Fig. 9.**
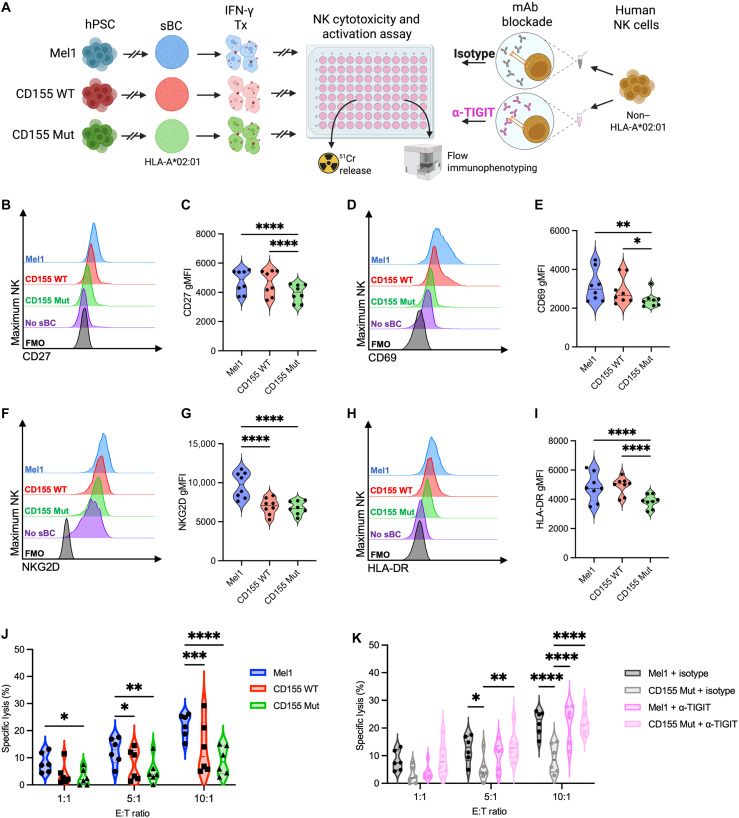
CD155 expression in sBC suppresses NK activation and NK-mediated cytotoxicity. (**A**) Experimental scheme depicting CML assay to assess the effect of TIGIT blockade (pink), relative to an isotype control (black) on CML of Mel1 (blue), CD155 WT–expressing (red), and CD155 Mut–expressing (green) sBC by NK cells, as measured by flow immunophenotyping of NK cells and ^51^Cr release assays [Created in BioRender. Brown, M. (2025); https://BioRender.com/2cr4baw]. (**B** to **I**) Violin plots show differences in expression of the NK cell activation markers [(B) and (C)] CD27, [(D) and (E)] CD69, [(F) and (G)] NKG2D, and [(H) and (I)] HLA-DR between sBC lines at the 1:1 E:T ratio compared to no sBC (purple) and no dye (black) controls. Data reflect biological *n* = 8 per condition. Significant *P* values are reported for one-way ANOVA with Bonferroni correction for multiple comparisons. (**J** and **K**) Violin plots show percent-specific lysis of (J) Mel1, CD155 WT, and CD155 Mut sBC at each E:T ratio in isotype control cultures and (K) Mel1 (dark gray and dark pink) or CD155 Mut (light gray and light pink) sBC at each E:T ratio with or without anti-TIGIT blockade. Data reflect biological *n* = 6 per condition. Significant *P* values are reported for three-way ANOVA with Bonferroni correction for multiple comparisons. *P < 0.05, **P < 0.01, ***P < 0.001, and ****P < 0.0001. mAb, monoclonal antibody.

In addition to T cell responses, innate immune recognition of transplanted β cells, especially in an allogeneic transplant context, can facilitate graft destruction. One innate immune subset that uses TIGIT signaling similarly to T cells is NK cells (*42*). As a result, we interrogated the ability of CD155-expressing sBC to inhibit NK activation and cell-mediated killing. HLA-mismatched NK cells were isolated from peripheral blood mononuclear cell (PBMC) donors followed by incubation with IFN-γ–treated Mel1, CD155 WT, or CD155 Mut sBC (Fig. 9A). The flow cytometry analysis of the NK cell activation markers CD27 (Fig. 9, B and C), CD69 (Fig. 9, D and E), NKG2D (Fig. 9, F and G), and HLA-DR (Fig. 9, H and I) displayed significant reductions in receptor expression gMFI after coculture within CD155 Mut sBC cocultures (CD27: 0.83-fold, *P* < 0.0001; CD69: 0.77-fold, *P* = 0.0013; NKG2D: 0.70-fold, *P* < 0.0001; HLA-DR: 0.79-fold, *P* < 0.0001) compared to Mel1 controls (*43*–*46*). Same as in T cell assays, CD155 WT–expressing sBC displayed a more intermediate suppressive impact, with significant reductions in NKG2D (0.72-fold, *P* < 0.0001) compared to Mel1 controls, while the other receptors’ expression was not significantly altered.

To determine the mechanistic role of CD155 expression on sBC within the NK cell assays, we preincubated NK cells with either an isotype control antibody or anti-TIGIT blocking antibody as in the T cell assays (Fig. 8). Chromium release assays quantifying sBC lysis after coculture experiments with the isotype control antibody revealed significant reductions in NK cell–mediated sBC lysis with CD155 Mut cocultures compared to Mel1 controls (1:1: 0.34-fold, *P* = 0.033; 5:1: 0.41-fold, *P* = 0.0029; 10:1: 0.41-fold, *P* < 0.0001) at all three E:T ratios (Fig. 9J). There were also significant reductions in specific lysis within CD155 WT sBC cocultures (5:1 0.49-fold, *P* = 0.047; 10:1: 0.62-fold, *P* = 0.0010) at the 5:1 and 10:1 ratios compared to Mel1 controls. Upon blockade of CD155:TIGIT engagement using the anti-TIGIT antibody, the Mel1 sBC cocultures displayed no significant differences compared to isotype antibody control cultures (Fig. 9K, dark gray and dark pink), mimicking our results within the T cell coculture experiments (Fig. 8F). However, within the CD155 Mut sBC cocultures, the blockade of TIGIT signaling significantly increased NK cell–mediated sBC lysis (5:1: 2.67-fold, *P* = 0.0038; 10:1: 2.47-fold, *P* < 0.0001) compared to the isotype control group (Fig. 9K, light gray and light pink). Collectively, these studies suggest that the expression of mutant CD155 on the surface of sBC has the ability to suppress both NK cell and T cell activation and sBC lysis, emphasizing the power of this system to inhibit both innate and adaptive immune responses.

## DISCUSSION

The development of durable, pancreatic β cell replacement therapies requires engineering strategies to enhance localized immune tolerance that can repress alloreactive and autoreactive T cell activation. The development of protocols to differentiate large pools of β cells from hPSC have improved the ability to generate immunomodulatory cell populations (*8*, *11*, *47*, *48*). Different approaches to accomplishing this goal have been attempted with mixed success. Preventing the expression of HLA molecules through deletion of Beta-2 Microglobulin (β*2M*) gene (HLA class I) or class II transactivator (*CIITA*) (HLA class II) genes proved attractive approaches to inhibit direct antigen presentation capabilities from the transplanted sBC (*17*–*19*, *49*). However, a major limitation to this approach is HLA class I^−/−^ cells are targeted by NK cells through the “missing self” response (*50*, *51*). The retention of HLA-E or HLA-G or the expression of CD47 can limit the NK-mediated destruction of engineered cells (*17*, *52*). We have previously engineered the expression of the check point inhibitor PD-L1 on sBC and demonstrated a reduction in stimulation of autoreactive T cell avatars (*19*). However, β*2M* knockout cell products may be fundamentally vulnerable in the event of tumorigenesis or exposure to infection due to challenges in immune surveillance. Therefore, approaches that can retain HLA expression while still effectively inhibiting T cell responses may be more attractive.

In this study, we demonstrated that sBC can be engineered to express CD155 to preferentially promote T cell coinhibitory signaling through the CD155/TIGIT axis and restrain both NK cell and T cell activation. Notably, we demonstrate that compared to a WT CD155 variant, a higher-affinity mutant CD155 confers even greater protection from NK cell and antigen-specific T cell killing by reducing immune cell effector function. Our findings provide compelling evidence that CD155 modulation may serve as an effective immune evasion strategy to bolster long-term graft survival for β cell replacement therapies in T1D. We have previously shown that the T1D risk-associated CD226/TIGIT axis can be modulated to reduce autoreactive T cell responses by inhibiting costimulatory CD226 signaling; however, we demonstrate that this pathway can also be modulated by augmenting coinhibitory TIGIT signaling to restrain autoimmunity and protect sBC. Furthermore, our data confirm that the membrane-bound CD155 Mut–expressing cells exhibit a higher binding efficiency for CD226 and an even greater binding for TIGIT compared to CD155 WT–expressing cells, as predicted by Matsuo *et al.* (*34*).

Chimienti *et al.* (*53*) have demonstrated that the genetic deletion of *CD155* in sBC in the context of HLA-I deficiency demonstrated the ability to circumvent the NK cell–targeted destruction. Hence, we hypothesize that the modulation of the CD155/TIGIT/CD226 axis in either direction—eliminating CD226:CD155 co-stimulatory signaling or bolstering TIGIT:CD155 coinhibitory signaling—may enhance the protection of sBC from adaptive immune responses. To our knowledge, this is the first study to express CD155 on the surface of sBC, demonstrating its success as an approach to diminish NK cell and antigen-specific T cell activation through TIGIT-mediated coinhibitory signaling. We also observed the ability for CD155-expressing sBC to suppress control antigen (MART-1) TCR avatars and polyclonal, allogeneic T cell proliferation, suggesting that this mechanism may inhibit broad T cell engagement at the localized graft site following transplantation. We corroborated that the enhanced protection previously conferred to CD155 Mut sBC was lost with the inclusion of an anti-TIGIT blocking antibody, emphasizing the role of CD155/TIGIT interactions in modulating immune responses (*34*). Notably, we did not observe the CD155 Mut sBC exhibiting greater susceptibility to CML from CD226:CD155 signaling during TIGIT blockade, relative to the Mel1 sBC, suggesting that CD226:CD155 signaling is not the driving signaling mechanism behind effector action in this system. Our group has recently demonstrated that inhibiting CD226 costimulatory signaling through anti-CD226 blocking antibodies inhibits murine effector T cell responses (*28*). Therefore, we would hypothesize that anti-CD226 treatment may yield additional protection to CD155-expressing sBC; however, additional studies of CD226:CD155 interactions in human sBC populations are needed.

Future studies should seek to assess the long-term and in vivo efficacy of engineering sBC to express immunomodulatory receptors, including whether CD155 engineering can be combined with other immunomodulatory approaches to enhance immune tolerance or T_reg_ recruitment. In addition, future preclinical studies should determine whether the protection conferred by CD155 expression differs as a result of TCR affinity, as our study used 1E6 TCR avatars, which exhibit a lower TCR:pMHC affinity resemblant of the typical affinity of autoreactive T cells found in vivo (*54*). While impossible to assess accurately in existing preclinical models, more interrogation into the ability for CD155 expression to inhibit the polyclonal nature of a human in vivo immune response that includes both allogeneic and recurrent autoimmune responses is also necessary to determine the potential effectiveness in clinical practice. For this to become a reality, better model systems that can recapitulate the human autoimmune interaction with sBC are required (*55*, *56*). Overall, our data support the continued investigation of engineering the expression of immunomodulatory molecules on sBC as a therapeutic strategy for enhancing the immune escape of sBC from innate immune and antigen-specific autoimmune T cell responses.

## MATERIALS AND METHODS

### Generation of WT and mutant CD155 constructs

CD155 plasmid template was purchased from OriGene (RC202254), followed by transformation, amplification, and purification in New England Biolabs (NEB) 5-alpha competent *Escherichia coli.* To generate the Ala^67^Thr mutant version of CD155, Q5 site-directed mutagenesis (NEB, E0554S) was performed, followed by transformation, amplification, and purification as with the original template. The confirmation of the mutation was performed through Sanger sequencing. Both WT and Mut CD155 constructs were then amplified through PCR and ligated into a targeting backbone (made in-house) with homology arms for the endogenous AAVS1 locus, neomycin resistance gene, and a CAG promoter before nucleofection into hPSC (described below). All primers used are listed in table S1.

### hPSC culture and TALEN-mediated engineering of CD155 WT and CD155 Mut lines

The undifferentiated hPSC Mel1^INS-GFP^ line [National Institutes of Health (NIH) registry #0139] (*35*) was dissociated into single cells using TrypLE incubation at 37°C for 6 min. Digestion was then quenched with mTeSR^+^ media, and live cells were counted using a Countess 3 cell counter (Thermo Fisher Scientific). Cells (2 × 10^6^) were transferred into microcentrifuge tubes and washed with phosphate-buffered saline (PBS). Washed cells were then prepared for nucleofection of TALEN-mediated knock-in (KI) of either a WT CD155 or Ala^67^Thr point-mutated (Mut) CD155 gene under control of a CAG promoter into the endogenous AAVS1 locus (fig. S1, A and B). Cells were nucleofected in P3 buffer per the Amaxa P3 Primary cell 4D-Nucleofector kit protocol (V4XP-3024) using the CB-150 program. AAVS1-TALEN-L, AAVS1-TALEN-R (Addgene, plasmid #59025 and 59026), and CAG-CD155 expression plasmids (generated in-house) were nucleofected into hPSC Mel1^INS-GFP^ cells. Nucleofected cells were then plated in 10-cm plates with 10 μM ROCK inhibitor (Y-27632, R&D Systems, #1254-50) and SCR7 (Xcess Biosciences, #M60082). After 24 hours, neomycin selection (50 μg/ml) was performed for 6 days. Remaining colonies were picked and expanded for further characterization. Genomic DNA was extracted from targeted colonies, and PCR analysis (fig. S1C and table S1) and flow cytometry for TALEN KI were performed to identify successfully modified clones. Once edited clones of CD155 WT– and CD155 Mut–expressing hPSCs were identified, the predominant clone used for the majority of further studies and the parental Mel1 hPSC line were sent for karyotyping via G-banding analysis (UF Health Pathology Cytogenetics Core). All submitted samples came back karyotypically normal (fig. S1, D to F).

### sBC differentiation

hPSC Mel1^INS-GFP^, CD155 WT, or CD155 Mut stem cell lines were maintained on human embryonic stem cell qualified Cultrex (Biotechne, #3434-005-002) in mTeSR^+^ media (STEMCELL Technologies, #05826). The differentiation to sBC was carried out in a suspension-based, magnetic stirring system (REPROCELL, #ABBWVS03A-6, #ABBWVDW-1013, #ABBWBP03N0S-6), as previously described (*9*, *19*, *20*). Briefly, 90% of confluent hPSC cultures were dissociated into single-cell suspensions by incubation with TrypLE for 6 min (Gibco, #12-604-021). Live cells were counted using a Countess 3 cell counter (Thermo Fisher Scientific), followed by seeding 0.5 × 10^6^ cells/ml in mTeSR^+^ media supplemented with 10 μM ROCK inhibitor in bioreactors. Three-dimensional cluster formation was performed for 48 hours, followed by washing of clusters with RPMI twice and induction of definitive endoderm (DE) differentiation using d1 media [RPMI containing 0.2% fetal bovine serum (FBS), 1:5000 insulin-transferrin-selenium (ITS) (Gibco, #41400-045), activin A (200 ng/ml; R&D Systems, #338-AC-01 M), and 3 μM CHIR99021 (STEMCELL Technologies, #72054)]. The differentiation medium was changed daily by letting clusters settle by gravity for 3 to 10 min. Most supernatant was removed by aspiration; fresh medium was added, and stirrer flasks were placed back on the system. sBC differentiation was based on our published protocol (*57*) with modifications as outlined below. Differentiation medias are as: days 2 and 3: RPMI containing 0.2% FBS, 1:2000 ITS, and activin A (100 ng/ml); days 4 and 5: RPMI containing 2% FBS, 1:1000 ITS, and keratinocyte growth factor (KGF) (50 ng/ml; PeproTech, #100-19-1MG); day 6: Dulbecco’s modified Eagle’s medium (DMEM) with d-glucose (4.5 g/liter; Gibco #11960-044) containing 1:50 N-21 MAX (Biotechne, #AR008), 1:100 nonessential amino acid (NEAA; Gibco, #11140-050), 1 mM sodium pyruvate (Gibco, #11360-070), 1:100 GlutaMAX (Gibco, #35050-061), 3 nM 4-[(E)-2-(5,6,7,8-Tetrahydro-5,5,8,8-tetramethyl-2-naphthalenyl)-1-propenyl]benzoic acid (TTNPB), (R&D Systems #0761), 250 nM Sant-1 (R&D Systems, #1974), 250 nM LDN193189 dihydrochloride (LDN) (STEMCELL Technologies, #72149), 30 nM phorbol 12-myristate 13-acetate (Sigma-Aldrich, #P1585-1MG), and 2-phospho-l-ascorbic acid trisodium salt (vitamin C) (50 μg/ml; Sigma-Aldrich, #49752-10G); day 7: DMEM containing 1:50 N-21 MAX, 1:100 NEAA, 1 mM sodium pyruvate, 1:100 GlutaMAX, 3 nM TTNPB, and vitamin C (50 μg/ml); days 8 and 9: DMEM containing 1:50 N-21 MAX, 1:100 NEAA, 1 mM sodium pyruvate, 1:100 GlutaMAX, epidermal growth factor (100 ng/ml; R&D Systems, #236-EG-01 M), KGF (50 ng/ml), and vitamin C (50 μg/ml); days 10 to 15: DMEM containing 1:50 N-21 MAX, 1:100 NEAA, 1 mM sodium pyruvate, 1:100 GlutaMAX, heparin (10 μg/ml; Sigma-Aldrich, #H3149-250KU), 2 mM *N*-acetyl-l-cysteine (Cysteine) (Sigma-Aldrich, #A9165-25G), 10 μM zinc sulfate heptahydrate (Zinc) (100 g; Sigma-Aldrich, #Z0251), 1× β-Mercaptoethanol (BME), 10 μM Alk5i II RepSox (R&D Systems, #3742/50), 1 μM 3,3′,5-triiodo-l-thyronine sodium salt (T3) (Sigma-Aldrich, #T6397), 0.5 μM LDN, 1 μM gamma secretase inhibitor XX (XXi) (AsisChem, #ASIS-0149), and 1:250 1 M NaOH to adjust pH to ~7.4; days 16 to 30: CMRL (Gibco, #11530-037) containing 1:50 N-21 MAX, 1:100 NEAA, 1:100 GlutaMAX, heparin (10 μg/ml), 2 mM cysteine, 10 μM zinc, 1x BME, 1 μM T3, 10 μM Alk5i II RepSox, vitamin C (50 μg/ml), 1:1000 trace elements A (Corning, #25-021-CI), 1:1000 trace elements B (Corning, #25-022-CI), and 1:250 NaOH to adjust pH to ~7.4. All media also contained 1× penicillin-streptomycin.

### Cryopreservation and thawing of sBC

At day 23, sBC were dissociated into single cells and cryopreserved as described (*9*, *19*). Briefly, clusters were digested and filtered using a cell strainer into fluorescence-activated cell sorting (FACS) 5-ml tubes. Cells were counted and resuspended at 3 x 10^6^ cells/100 μL of CryoStor CS10 (StemCell Technologies). Cells were placed in cryovials (100 μl per vial), transferred into precooled freezer buddies, and placed into the −80°C overnight before transfer to liquid nitrogen for long-term storage. For thawing, frozen vials of sBC were placed in a 37°C bead bath for 3 min to warm. Then, 1 ml of warm sBC media (days 16 to 30 media described above) was added to the cryovial dropwise before the entire volume was transferred into 5 ml of sBC media in one well of a six-well suspension plate to create clusters and placed in the 37°C incubator on an orbital shaker set at 95 rpm or seeded as single cells for sequential T cell assays.

### Dynamic GSIS assay

Dynamic insulin secretion was measured using a BioRep Technologies perifusion machine (PERI4-115-1810-076). 50 sBC clusters were placed on a filter in the perifusion chamber, and various solutions were perfused through the system at 100 μl/min by a peristaltic pump; cells and solutions were kept at 37°C and ambient atmosphere. The perifusion program consisted of a 30-minute preincubation with KRB buffer containing low (2.8 mM) glucose followed by the following run program: (i) 30 min low glucose, 20 min high (16.7 mM) glucose, 20 min high glucose + IBMX (50 μM; MillaporeSigma), 15 min low glucose, 5 min KCl (30 mM), 10 min low glucose. Perifusion flow-through was collected in 96-well plates and stored at 4°C overnight or − 20°C if longer storage was needed for future analysis. Cell pellets were recovered, lysed with acid/ethanol solution, and frozen overnight for assessment of total insulin content using a human insulin enzyme-linked immunosorbent assay (ALPCO, 80-INSHU-E10.1).

### IF imaging of sBC

Thawed sBC clusters were collected in 1.5-ml Eppendorf tubes and allowed to gravity settle for 5 min. Clusters were washed with 1× PBS and fixed for 10 min in 4% paraformaldehyde, blocked/permeabilized with CAS block buffer containing 0.4% Triton X-100 (CAS-T) for 10 min, and stained with primary antibodies overnight at 4°C in CAS-T buffer (table S2). Clusters were washed and stained with fluorochrome-conjugated secondary antibodies for 2 hours at room temperature in CAS-T buffer, mounted on microscope slides (VWR) with ProLong Gold antifade reagent with 4′,6-diamidino-2-phenylindole (Invitrogen), and sealed with cover slips. Z-stack images were collected on Zeiss confocal microscope (LSM 710), followed by maximum intensity projection (Zeiss Black software).

### Human T cell and NK cell subjects

Fresh PBMCs were obtained from human leukapheresis-enriched blood of deidentified, Institutional Review Board (IRB)-exempt, healthy donors (T cell donors: median age: 24 years, range 19 to 43 years, *N* = 11, 63.6% female; NK cell donors: median age: 33.5 years, range 18 to 49 years, *N* = 4, 75% female) purchased from LifeSouth Community Blood Centers (Gainesville, FL, USA).

### Packaging and titration of LV vectors

The MART-1 (melanoma antigen-specific) TCR (*58*) and 1E6 (pre-proinsulin, PPI-specific)–TCR (*59*) lentiviral (LV) constructs were packaged in third-generation lentivirus vectors containing a GFP or Rat-CD2 reporter, respectively, produced by human embryonic kidney–293 cells cotransfected with the vesicular stomatitis virus G envelope and Gag/Pol and Rev packaging plasmids, as previously described (*60*). Forty-eight hours after transfection, viral supernatant was collected, centrifuged, and filtered before concentrating with polyethylene glycol (VectorBuilder, Chicago, IL, USA). The infectivity of each vector was quantified as infectious units/ml (IFU/ml) by establishing the frequency of GFP^+^ or Rat-CD2^+^ cells compared to the volume of LV supernatant used. Subsequent transductions were performed at a concentration of 3 transducing units (TU)/cell, where 1 IFU = 1 TU.

### LV transduction and expansion of CD8^+^ T cells

Naïve CD8^+^ T cells were isolated from whole PBMCs using the EasySep Human Naïve CD8^+^ T Cell Isolation Kit II (STEMCELL Technologies, Vancouver, BC, Canada) and plated at 2.5 × 10^5^ cells per well in 1 ml of complete RPMI media [RPMI 1640 media phenol red without l-glutamine 139 (Lonza, Basel, CH-BS, Switzerland), 5 mM Hepes (Gibco, Waltham, MA, USA), 5 mM minimum essential medium 140 NEAA (Gibco), 2 mM GlutaMAX (Gibco), penicillin 141 (50 μg/ml; Gibco), streptomycin (50 μg/ml; Gibco), 20 mM sodium pyruvate (Gibco), 50 mM 2-mercaptoethanol (Sigma-Aldrich, St. Louis, MO, USA), 20 mM sodium hydroxide (Sigma-Aldrich), and 10% FBS (Genesee Scientific, El Cajon, CA, USA)]. CD8^+^ T cells received recombinant human interleukin-2 (rhIL-2; 100 IU/ml) and were stimulated with Dynabeads Human T-Expander CD3/CD28 (Thermo Fisher Scientific, Waltham, MA, USA) at a 1:1 bead:cell ratio. After 48 hours of stimulation, cells were treated with protamine sulfate (8 μg/ml) and transduced with 3 TU per cell of the corresponding LV vector before spinnoculation (1000*g*, 30 min at 32°C). Cell culture medium was changed every 2 to 3 days, and beads were removed on day nine. To increase the purity of successfully transduced T cell “avatars” for coculture experiments, FACS was used to enrich GFP^+^ or Rat-CD2^+^ cells after expansion with a BD FACSMelody Cell Sorter (BD Biosciences, Franklin Lakes, NJ, USA).

### Human NK cell isolation

To verify that potential NK cell donors lacked the HLA-A2 serotype, 50,000 PBMCs were used for flow cytometry. Fc receptors were blocked using TruStain FcX (BioLegend, RRID: AB_2818986) for 5 min at 23°C before staining with an HLA-A2-Pacific Blue antibody (clone, RRID, concentration, and manufacturer information provided in table S2) for 15 min at 23°C. Cells were washed once with stain buffer [PBS + 2% FBS + 0.05% NaN_3_ (w/v)] before analysis. Data were collected on an Aurora 5L (16UV-16 V-14B-10YG-8R) spectral flow cytometer (Cytek, Freemont, CA, USA) and analyzed using FlowJo software (TreeStar, version 10.8.1). Using donors without the HLA-A2 serotype, human primary NK cells were isolated from whole PBMCs using the EasySep Human NK Isolation Kit (STEMCELL Technologies) for subsequent experiments.

### Immunoglobulin binding assay

Recombinant human TIGIT-Immunoglobulin (TIGIT-Ig; BioLegend, San Diego, CA, USA) and CD226-immunoglobulin (CD226-Ig; BioLegend) chimeric proteins were fluorescently labeled using a Zenon Alexa Fluor 594 (AF-594) human IgG labeling kit (Thermo Fisher Scientific) according to the manufacturer’s protocol, yielding a final concentration of 88 ng/μL for AF-594–labeled TIGIT-Ig and AF-594–labeled CD226-Ig. A total of 50,000 cells were stained with LIVE/DEAD Near-IR viability dye (Invitrogen, Waltham, MA, USA) for 10 min at 4°C before washing with stain buffer. Cells were resuspended at a concentration of 2.5 × 10^5^ cells per ml in 1× PBS (Gibco) and treated with AF-594–labeled TIGIT-Ig or AF-594–labeled CD226-Ig for 30 min at 37°C at the following concentrations: 0, 1, 10, 100, 50, 1000, 2500, 5000, 10,000, and 25,000 ng/ml. Unbound TIGIT-Ig or CD226-Ig was removed by washing cells with stain buffer. Flow cytometry data were collected and analyzed as described above.

### sBC flow cytometry phenotyping

sBC were pretreated with or without IFN-γ (100 ng/ml) for 2 days. A total of 2 × 10^5^ cells from each condition were used for flow cytometry and were stained with LIVE/DEAD Near-IR viability dye as described above. Cells were stained with an extracellular antibody cocktail consisting of anti-human CD112–phycoerythrin (PE)/Cyanine7, CD155-Alexa Fluor 647, CD274-BV711, HLA-A2-Pacific Blue, and HLA-A,B,C–PE for 30 min at 23°C (clone, RRID, concentration, and manufacturer information provided in table S2). Flow cytometry data were collected and analyzed as described above. Gating strategies were determined using fluorescence-minus one (FMO) and unstained controls.

### T cell and NK cell activation assay

To assess T cell or NK cell activation by flow cytometry after coculture with sBC, 30,000 single sBC were plated on Cultrex-coated wells in a 96-well plate in days 16 to 23 media (described above), followed by 48 hours of IFN-γ treatment (100 ng/ml). sBC were then washed with complete DMEM (cDMEM) media (DMEM; Lonza), 5 mM Hepes (Gibco), 5 mM MEM NEAA (Gibco), penicillin 141 (50 μg/ml; Gibco), streptomycin (50 μg/ml; Gibco), 0.02% bovine serum albumin (Sigma-Aldrich), and 10% FBS (Genesee Scientific) and cocultured with MART-1 or PPI-reactive (1E6) T cell avatars at 10:1 E:T ratios for 48 hours or with primary NK cells at a 1:1 E:T ratio for 24 or 48 hours. After coculture, immune cells were harvested and stained with LIVE/DEAD Near-IR viability dye as described above. Following this, Fc receptors were blocked using TruStain FcX (BioLegend, RRID: AB_2818986) for 5 min at 23°C before extracellular staining in the presence of Brilliant Stain Buffer Plus (BD Biosciences) with panels for either T cell (CD8-AF700, CD96-BV421, CD226-PE-Cy7, and TIGIT-PerCP eFluor710) or NK cell (CD3-AF700, CD27-Pacific Blue, CD56-SparkPLUS UV395, CD69-BV711, CD314-PE, and HLA-DR-BV570) markers of interest for 30 min at 23°C (clone, RRID, concentration, and manufacturer information provided in table S2). Flow cytometry data were collected and analyzed as described above. Gating strategies were determined using FMO and unstained controls.

### Proliferation assay

To assess the immunogenicity of each sBC line toward initiating an allogeneic, proliferative response in polyclonal naïve CD8^+^ T cells, we performed T cell proliferation assays. Briefly, naïve CD8^+^ T cells were isolated, as described above, and labeled with CellTrace Violet (Thermo Fisher Scientific, catalog no. C34557A) proliferation tracking dye, as recommended by the manufacturer’s protocol. Subsequently, 187,500 labeled naïve CD8^+^ T cells were cultured at a 1:1 ratio with 2-day IFN-γ pretreated sBC for each condition in a 24-well plate and supplemented with rhIL-2 at 100 IU/ml. After 6 days of coculture, suspension cells were collected and underwent viability staining with LIVE/DEAD Near-IR viability dye and Fc receptor blocking, as described above before staining with CD8-AF700 for 30 min at 23°C (clone, RRID, concentration, and manufacturer information provided in table S2). Flow cytometry data were collected and analyzed using proliferation modeling to determine the proliferation index using FlowJo software, as described above.

### Chromium release assay

The susceptibility of sBC lines to CML by T cell avatars and primary NK cells was assessed using chromium release assays in the format previously described by Chen *et al.* (*61*). sBC were plated and treated with IFN-γ as described above before radiolabeling with ^51^CrNa_2_O_4_ (Revvity, Waltham, MA, USA) at an activity of 1.48 × 10^5^ Bq per well for 4 hours before washing twice with fresh cDMEM. sBC were cocultured with MART-1 or PPI-reactive (1E6) T cell avatars at 0:1, 1:1, 5:1, and 10:1 E:T ratios for 48 hours or with primary human NK cells for 24 hours.

For chromium release assays involving TIGIT blockade, PPI-reactive T cells or primary NK cells were concentrated at 3 × 10^6^ cells/ml cDMEM and incubated for 30 min at 37°C on an orbital shaker in the presence of either mouse IgG2b, kappa isotype control (BioLegend; RRID: AB_2744505) or anti-human TIGIT mAb (40 μg/ml) (BioLegend; RRID: AB_2820102) before washing into fresh cDMEM and plating as described above.

Following coculture, the supernatants were removed, and along with 200 μl of 1× PBS used to wash the wells, and were transferred into 6 mm–by–50 mm lime glass tubes. The lysates of adherent cells were collected using a 2% SDS wash and transferred into separate tubes. ^51^Cr activity, measured in counts per minute (CPM), was assessed for both fractions on a Wizard 1470 automatic gamma counter (Revvity). The specific lysis of sBC was calculated as follows $\%\text{Specific lysis}=\text{Experimental}\;\left[\frac{\left(\text{CPM}\;\text{of supernatant}\right)}{\left(\text{CPM}\;\text{of supernatant}\right)\;+\;\left(\text{CPM}\;\text{of lysate}\right)}\right]-\text{Spontaneous}\;\left[\frac{\left(\text{CPM}\;\text{of supernatant}\right)}{\left(\text{CPM}\;\text{of supernatant}\right)\;+\;\left(\text{CPM}\;\text{of lysate}\right)}\right]$

### Cytokine multiplex assay

To quantify the production of cytokines by T cells after coculture with sBC, cell culture supernatants from the CML assay were used to evaluate IL-2, IL-4, IL-6, IL-10, IL-17A, FasL, IFN-γ, tumor necrosis factor (TNF), granzyme A, granzyme B, perforin, and granulysin production. Using the LEGENDplex Human CD8/NK Panel Kit (BioLegend), the production of IFN-γ and granzyme A were measured at a 1:100 dilution, whereas FasL, granzyme B, perforin, and granulysin were measured at a 1:1 dilution, with IL-2, IL-4, IL-6, IL-10, IL-17A, and TNF not detected in the assay using a Aurora 5L spectral flow cytometer. Data were analyzed using the LEGENDplex Data Analysis Software Suite (version 2024-09-10; BioLegend). Dilution factors and analyte detection ranges are described in table S3.

### Data visualization and statistical analysis

Statistical analyses were performed using GraphPad Prism software (version 10.3.1; San Diego, CA, USA). Unless otherwise stated, chromium release and flow cytometric data were analyzed by two-way analysis of variance (ANOVA), with Bonferroni’s post hoc test for multiple testing correction. Proliferation indices and binding area under the curve values were analyzed using one-way ANOVA with Bonferroni’s post hoc test for multiple testing correction. *P* values ≤ 0.05 were considered significant.
